# Alterations of oncogenes expression in NK cells in patients with cancer

**DOI:** 10.1002/iid3.179

**Published:** 2017-07-10

**Authors:** Gulnur K. Zakiryanova, Elena Kustova, Nataliya T. Urazalieva, Aday Amirbekov, Emile T. Baimuchametov, Narymzhan N. Nakisbekov, Michael R. Shurin

**Affiliations:** ^1^ Scientific and Technological Park Al‐Farabi Kazakh National University Almaty Kazakhstan; ^2^ Laboratory of Immunology Scientific Center of Pediatric and Children Surgery Almaty Kazakhstan; ^3^ Joint Use Center, Atchabarov Scientific‐research institute of fundamental and applied medicine Asfendiyarov Kazakh National Medical University Almaty Kazakhstan; ^4^ Kazakh Research Institute of Oncology & Radiology Almaty Kazakhstan; ^5^ Clinical Immunopathology University of Pittsburgh Medical Center Pittsburgh Pennsylvania

**Keywords:** c‐Kit, c‐Myc, mbSCF, sSCF, STAT3

## Abstract

**Introduction:**

C‐kit/SCF signaling plays a key role in regulating NK cell homeostasis, maturation, proliferation, and cytotoxicity. C‐kit‐deficiency in NK cells results in significant reduction of their number, suggesting an imperative role for c‐kit signaling in NK cell biology. We have recently showed that human NK cells express not only c‐kit‐receptor, but also both membrane‐bound and soluble forms of c‐kit ligand—Stem cell factor. The goal of this study was to characterize the c‐kit/SCF autocrine loop in peripheral blood NK cells obtained from patients with cancer.

**Methods:**

Using Smart Flare and qRT‐PCR, we have characterized expression of c‐kit and two forms of SCF in patients’ NK cells and correlated these results with the expression of c‐myc and STAT3.

**Results:**

Our results demonstrated that the expression of proto‐oncogenes c‐myc and c‐kit was significantly decreased in NK cells from all cancer patients. Expression of membrane‐bound SCF in NK cells correlated with the presence of remote metastases.

**Conclusions:**

We suggest that the abnormal signaling and expression of c‐kit/SCF, c‐myc, and STAT3 in NK cells is responsible for the defect in their cytolytic activity in cancer and these defects at the gene expression level may be the cause rather than the result of tumor progression.

## Introduction

NK cells play a crucial role in antitumor immunity by direct killing of malignant cells and releasing a number of cytokines that regulate both innate and adaptive immune responses. Normal NK cells display a strong cytotoxic activity, which, however, may be diminished during tumor development. In fact, profound impairment of NK cell activity has been demonstrated in cancer patients, including decreased cytotoxicity, defective expression of activating receptors, or intracellular signaling molecules, overexpression of inhibitory receptors, abnormal proliferation, decreased tumor infiltration, and defective cytokine production 1, 2. Maturation defects of human NK cells in the peripheral blood of cancer patients and in the bone marrow in tumor‐bearing mice models have also been reported 3, 4. However, the intracellular mechanisms associated with abnormal NK cell function in cancer are not well characterized.

The stem cell factor/c‐Kit signaling pathway is pivotal for the survival, differentiation, and maturation of NK cells 5. SCF together with IL‐15 mediate differentiation of human NK cells from CD34+ hematopoietic progenitors, which is associated with the synthesis and lytic activity of the key cytotoxic proteins—catepsin C, granzyme B, and perforin 6. The defect of SCF/c‐kit signaling pathway has been described in many tumors. As a member of the tyrosine kinase family, c‐kit receptor signaling regulates expression of certain genes, modulates vital function of many cell populations, and plays an important role in tumor occurrence, development, and spreading 7. However, in spite of reported importance of SCF/c‐kit signaling for NK cell function and frequent malfunction of this signaling pathway during tumor development, the analysis of с‐kit and SCF expression in NK cells in cancer has not been yet carried out.

The c‐myc gene was identified based on the homology with the retroviral oncogene, and the c‐myc proto‐oncogene was shown to be activated in many animal and human tumors 8. The c‐*myc* gene product, a transcription factor, regulates a variety of cellular processes involved in cell growth, proliferation, apoptosis as well as cellular metabolism 9. C‐myc is involved in IL‐15 signaling pathway, which is critical for NK cell maturation and homeostasis 10. In fact, it has been reported that the overexpression of c‐Myc during NK cell development contributes to the overall transcription of multiple *KIR* (the killer cell immunoglobulin‐like receptor) genes. Together with the fact that binding of endogenous c‐Myc to the distal promoter element is significantly enhanced upon IL‐15 stimulation in peripheral blood NK cells and correlates with an increase in *KIR* transcription, this provides a direct link between NK cell activation signals and KIR expression required for acquisition of the effector function during NK cell education 11. In addition, it has been demonstrated that stimulation with IL‐2, an important regulator of NK cell activity, increases c‐myc expression in natural killer cell line NK3.3 12. However, c‐myc expression in NK cells in cancer patients has never been evaluated.

Signal transducers and activators of transcription (STAT) protein STAT‐3 performs a key role in mediating signaling by c‐kit and c‐myc. In fact, the signal transduction pathway from the PDGF receptor (c‐kit is member of RTK class III—PDGF receptor family) to the nucleus results in signaling to STAT‐3, which, in turn, induces the expression of c‐myc 13, 14. It is known that NKG2D expression in NK cells is regulated at the transcriptional level by STAT‐3, resulting in a functional NK cell defect in patients with STAT‐3 mutations 15. STAT‐3 is involved in driving the most pathways that control NK cytolytic activity as well as the reciprocal regulatory interactions between NK cells and other components of the immune system 16.

Here, we determined the c‐myc, с‐kit, membrane‐bound SCF (mbSCF) and soluble SCF (sSCF) and STAT3 expression in NK cells in patients with different types of cancer. Our results revealed a strongly declined expression of oncogenes c‐myc and c‐kit, while STAT‐3 expression in contrary was increased in NK cells from lung cancer patients but was down‐regulated in NK cells from gastric, sigmoid, and colon cancer patients. Expression of mbSCF in NK cells correlated with the presence of remote metastasis. These clinical data add new insights in our understanding of NK cell immunobiology in cancer and may provide new targets for NK cell‐based immunotherapeutic approaches to cancer treatment.

## Materials and Methods

### Patients and samples

Peripheral blood specimens were collected from 28 patients (median age 62, [53–79]) with different types of cancer, including lung cancer (adenocarcinoma, squamous cell carcinoma, small cell lung cancer [SCLC]), bladder adenocarcinoma, esophageal adenocarcinoma, colorectal cancer, gastric cancer, and sigmoid cancer (Table 1). All patients gave their informed written consent for participation in this study, which was reviewed and approved by the Institute of Oncology & Radiology, (Almaty, Kazakhstan) IRB committee in line with the Declaration of Helsinki. Blood was collected prior to the surgical and chemotherapy procedures. Healthy controls (HC, *n* = 20, median age 30, [24–56]) were recruited from the personnel of the Laboratories within the B. Atshabar Research Institute of Fundamental and Applied Medicine after signing of the informed consent forms. It is believed that age does not have a significant effect on Myc expression in human lymphocytes as well as any mouse tissue. Moreover, we suppose that tumorogenesis is very long process that spanned decades and ages correspondence of control group and patients are not critical point.

**Table 1:** Patient characteristics

Characteristics	Total patients (*N* = 28)
Sex	%
Male	21 (75%)
Female	7 (30%)
Age	Years
Median	62
Range	53‐79
Prior anticancer therapies	No
Lung cancer:	14 (50%)
NSCLC	14 (50%)
Esophageal ADC	1(3.5%)
Cardio‐esophageal‐gastric ADC	1(3.5%)
Bladder ADC	1(3.5%)
Colorectal cancer	3 (10, 7%)
Sigmoid cancer	3 (10,7%)
Gastric ADC	4 (14%)
Stage of differentiation of gastric ADC:	
High	1 (3.5%)
Moderate	2(7%)
Low	1 (3.5%)
Metastasis: Primary tumor site	Localization of metastasis
Lung ADC 1	Brain and spinal cord
Lung ADC 2	Ribs
Colorectal cancer 2	Liver, lung
Bladder ADC 1	Lymph nodes

### Purification of NK cells: Isolation of c‐kit^pos^ and c‐kit^neg^, and CD56^bright^/CD56^dim^ NK cells

PBMC were isolated from the peripheral blood by Ficoll‐Paque™ PLUS (Life Technologies, Pittsburgh, PA, USA) density gradient centrifugation. NK cells were negatively selected using DynaMag™‐5 Magnet with Dynabeads® Untouched™ Human NK Cells isolation kit (Life Technologies, USA). The purity of the cell subsets was confirmed by flow cytometry (FACSCalibur, BD Biosciences, San Jose, CA, USA) using appropriate monoclonal antibodies labeled with FITC and PerCP (BD Biosciences). After negative selection of NK cells, c‐kit‐positive, and c‐kit‐negative subsets of NK cells were separated using human CD117 (anti‐c‐kit antibody) covered microbeads (Miltenyi Biotech, Germany). For CD56^bright^ and CD56^dim^ separation, CD56 + CD16 + NK Cell Isolation Kit was used. Separation was conducted according to the manufacturer's instructions (Myltenyi Biotec, Germany).

### Real‐time quantitative polymerase chain reaction

Reaction mixture consist of (i) Master Mix − 13.5 μl; (ii) PCR Mix—2.5 μl; (iii) cDNA (40 ng)—1.0 μl; (iv) Tag pol—1.0 μl, (v) MilliQ—2.0 μl. qRT‐PCR was performed using equal amounts of RNA isolated from CD117‐positive and CD117‐negative NK cell samples. Separated cells were washed with PBS, and the cell pellets were frozen in TRIzol™ Reagent (Life Technologies, USA). Total RNA was isolated according to the manufacturer's instructions. cDNA was then synthesized using the High‐Capacity RNA‐to‐cDNA™ Kit (ThermoFisher Scientific, Waltham, MA, USA). The qRT‐PCR amplification was performed with the following setting: 1 cycle at 95°C for 3 min; 45 cycles at 95^°^С for 10 s, 55^°^С for 40 s. The following primers used were: c‐kit, Fw (5‐GTCCTACGCTTCCAA‐3), and Rev (5‐ACTTCAATTATGAACGTC‐3); mbSCF set 1, Fw (5‐CTTTCCTTATGAAGAAGAC‐3), Rev (5‐TTATCCAACAATGACTTG −3); sSCF set 2, Fw (5‐CTTCCTATTACTGCTACT‐3), Rev (5‐TTCAACATTAAGTCCTGA‐3). The expression levels were calculated as 2^−ΔΔCt^, where relative expression was determined by normalization to the β‐actin gene expression.

### SmartFlare™ RNA detection assay

Isolated CD117‐positive and CD117‐negative NK cell samples were incubated in RPMI‐1640 medium supplemented with 10% FCS in 96‐well flat‐bottom plates at 37°C, 5% CO_2_ for 20 h in the presence of Smart Flare MYC, Human, Cyanine 5, Smart Flare KIT. Separated CD56^bright^ and CD56^dim^ NK cells were incubated in the presence of Smart Flare STAT3, Human, Cyanine 5 according to the manufacturer's instructions (Merck Millipore, Billerica, MA, USA). Control probes were incubated with: (i) Cellular uptake control; (ii) Scramble control for specificity; and (iii) Housekeeping control—the 18 s gene. Fluorescence was detected by flow cytometry on BD FACSCanto™ II.

### Statistical analysis

Mann–Whitney Rank Sum test was used to evaluate the statistical significance of differences between cancer patients and healthy donors. All results are expressed as the mean ± SEM. *p* value < 0.05 was considered significant.

## Results

### C‐kit expression in NK cells from cancer patients was significantly decreased independently of the tumor location

Detection of c‐kit expression in CD117(c‐kit)‐positive and ‐negative fractions of NK cells from cancer patients and healthy donors was carried by two methods: *qRT‐PCR* and Smart Flare. All data obtaining by qRT‐PCR were entirely confirmed by Smart Flare method, which allows detection and visualization of mRNA in live cells. Based on the facts that (i) CD56^bright^ population of NK cells is the only lymphocyte population in the peripheral blood with the constitutive expression of the c‐kit receptor 17; (ii) c‐kit expression gradually vanishes during NК differentiation from CD56^bright^ to CD56^dim^ cells 18; and (iii) c‐kit is responsible for proliferation and maturation of NK cells 19, 20, 21; these data allow the following suggestion: the tumor‐associated defects in NK cell maturation are connected with the ectopic expression of c‐kit and/or SCF not only in c‐kit‐positive, but also in c‐kit‐negative subset of NK cells. Therefore, to test this and to determine whether the formation of *SCF* and *c‐kit* transcripts is occurring in all NK cells or only in c‐kit‐positive NK cell subset in cancer patients, total NK cells were sorted for the c‐kit‐positive and c‐kit‐negative populations.

**Figure 1:**
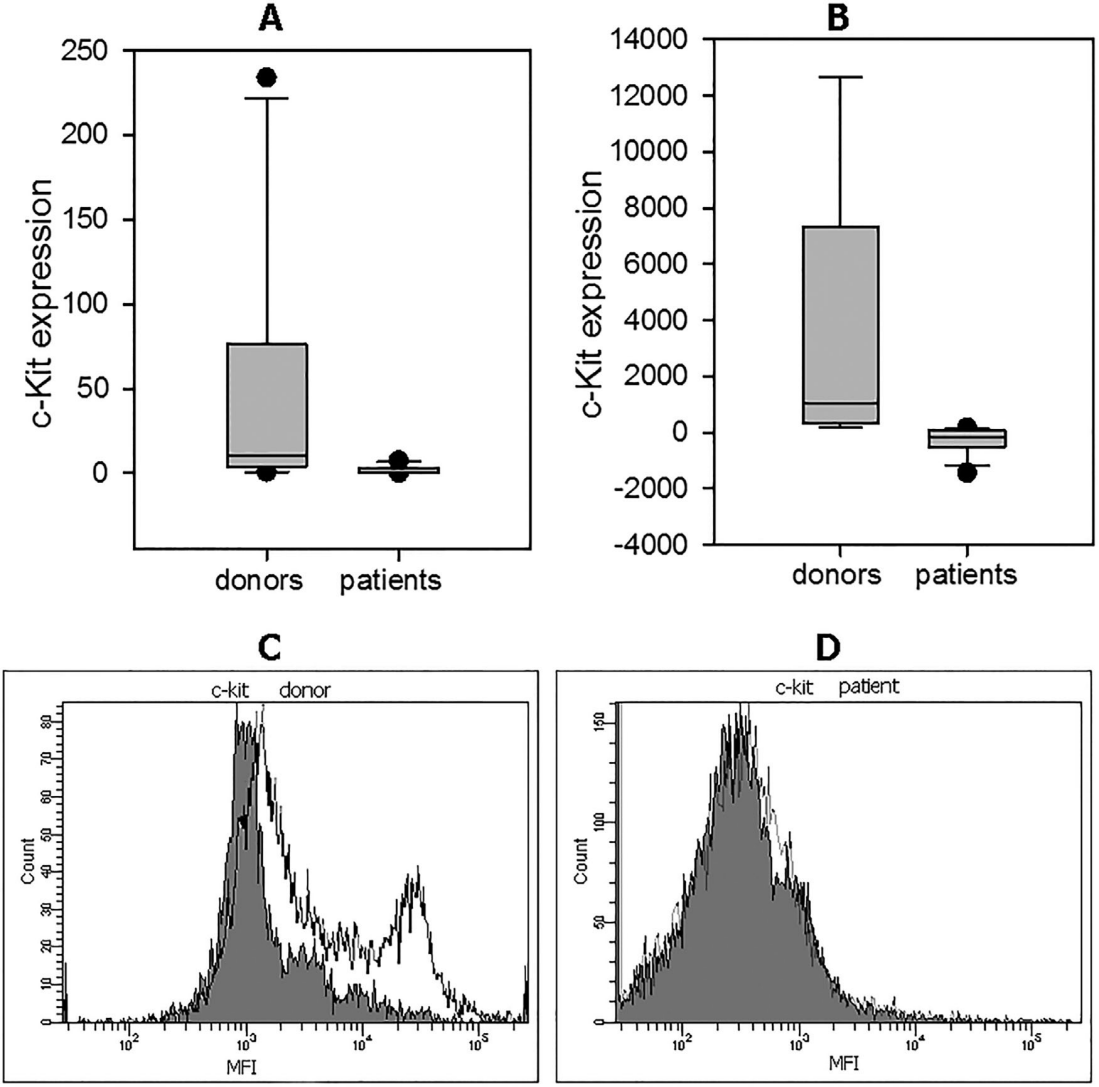
C‐kit expression in NK cells from patients with cancer and healthy donors. (A) C‐kit expression in CD117‐positive NK cells was assessed by qRT‐PCR as described in M&M. The expression levels were calculated as 2^− ΔΔCt^, where relative expression was determined by normalization to the beta‐actin gene expression. The mean ± SEM data for healthy donors and patients with cancer are shown. C‐kit expression by CD117‐positive NK cell subset in all patients was strongly decreased irrespective of the differentiation stage and disease severity: 47.5 ± 23.7 (*n* = 10) in healthy donors versus 1.57 ± 0.67 in patients (*n* = 12) with cancer (*p* < 0.001). (B) C‐kit expression in CD117‐positive NK cells was assessed by Smart Flare as described in M&M. NK cell samples were incubated in RPMI‐1640 medium supplemented with 10% FCS in 96‐well flat‐bottom plates at 37°C, 5% CO_2_ for 20 h in the presence of Smart Flare KIT, Human, Cyanine 5. The mean ± SEM data for healthy donors and patients with cancer are shown. MFI values were 267 ± 128 (*n* = 12) in patients with cancer versus 3427 ± 1510 in healthy donors (*n* = 9) (*p* < 0,001). (C) Representative Smart Flare results of C‐kit expression in CD117^+^ NK cells from one of 12 healthy donors (D) and one of nine cancer patients are shown. Gray histograms—is negative control, white—is positive expression.

The results revealed that the ratio of c‐kit‐positive to c‐kit‐negative NK cells was similar in cancer patients and healthy donors, although the total number of NK cells harvested from the peripheral blood of cancer patients was twice higher than NK cell numbers in the same volume of blood in healthy donor. For instance, from 10 ml of the peripheral blood of healthy donors we standardly harvested 1 × 10^6^ NK cells, while from the same quantity of patients’ blood, independently on the type and localization of cancer, we usually obtained 2 × 10^6^ NK cells. This allowed us to hypothesize that increased NK cell numbers in cancer patients may be associated with of altered expression of c‐kit in CD117‐positive NK cells. In fact, we revealed that c‐kit expression was significantly decreased in NK cells in cancer patients independently of cancer type. C‐kit expression by CD117‐positive NK cell subset in patients with non‐small lung cancer, cancer of gallbladder (moderately differentiated), cancer of esophagus (epidermoid type), colorectal cancer (infiltrated and squamous cancer, all with metastasis in lymph nodes or lungs or liver), and sigmoid cancer was strongly decreased irrespective of the differentiation stage and disease severity: 47.5 ± 23.7 (*n* = 10) in healthy donors versus 1.57 ± 0.67 in patients (*n* = 12) with cancer (*p* < 0.001, Fig. 1A).

Next, c‐kit expression in NK cells was significantly decreased in all patients. All data from the qRT‐PCR detection were confirmed by the results from the Smart Flare method. The difference in the median values between the two groups was statistically significant. For instance, MFI values were 267 ± 128 (*n* = 12) in patients with cancer versus 3427 ± 1510 in healthy donors (*n* = 9) (*p* < 0,001) (Fig. 1B). Finally, c‐kit expression in CD117(c‐kit)‐negative fraction of NK cells from all cancer patients was similar to c‐kit expression in CD117(c‐kit)‐negative fraction of NK cells in healthy donors and was technically undetectable.

Thus, our data demonstrate the lower expression of c‐kit in c‐kit‐positive subset of NK cells in patients with cancer.

### MbSCF overexpression in NK cells from cancer patients correlated with the presence of remote metastasis and disease severity

**Figure 2:**
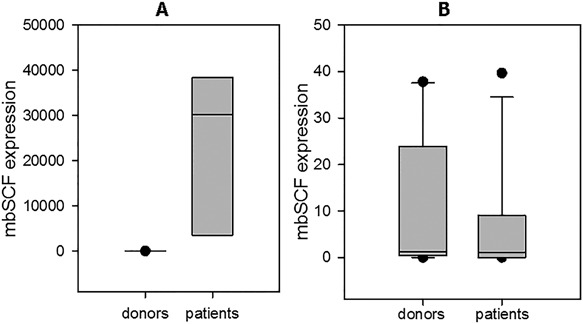
MbSCF expression in NK cells from patients with cancer and healthy donors. (A) MbSCF expression in CD117‐positive NK cells from patients with remote metastasis was assessed by qRT‐PCR as described in M&M. The expression levels were calculated as 2^ −ΔΔCt^, where relative expression was determined by normalization to the beta‐actin gene expression. Number of samples: cancer patients with remote metastasis (*n* = 3), healthy donors (*n* = 10). The mean ± SEM data for healthy donors and patients with cancer are shown. A total of 24,041 **± **10,525 in patients versus 9.9 **± **4.8 in healthy donors (*p* = 0.014). (B) MbSCF expression in CD117‐positive NK cells from patients without metastasis was assessed by qRT‐PCR as described in M&M. Number of samples: cancer patients without metastasis (*n* = 12), healthy donors (*n* = 10). The mean ± SEM data for healthy donors and patients with cancer are shown. mbSCF expression was 6.4 **± **3.6 in patients versus 9.9 **± **4.8 in healthy donors (*p* = 0.72).

We next determined the mbSCF expression in CD117(c‐kit)‐positive and ‐negative fractions of NK cells from cancer patients and donors by qRT‐PCR since Smart Flare for SCF is not yet available. In contrast to the c‐kit expression, mbSCF expression by CD117(c‐kit)‐positive and ‐negative fractions of NK cells from cancer patients and healthy donors was similar with the exception of three inoperable patients with remote metastasis, whose NK cells expressed extremely high levels of mbSCF. These patients include a patient with lung cancer and metastases in the brain and spinal cord, a patient with cardioesophageal cancer, and metastases in the surrounding tissues of esophagus and stomach and a patient with lung cancer on the disintegration stage: 24041 **± **10525 in patients (*n* = 3) versus 9.9 **± **4.8 in healthy donors (*n* = 10) (*p* = 0.014) (Fig. 2A). In the main group of patients without metastasis, mbSCF expression was 6.4 **± **3.6 in patients (*n* = 12) versus 9.9 **± **4.8 in healthy donors (*n* = 10) (*p* = 0.72) (Fig. 2B).

MbSCF expression in CD117(c‐kit)‐negative NK cells from all cancer patients was similar to that one in healthy donors and was technically undetectable.

Thus, mbSCF expression, in contrast to c‐kit expression, correlated with the metastatic spread of malignant cells, which can be connected with the role of mbSCF in the regulation of cell mobility.

### sSCF expression in CD117‐positive NK cell subset from cancer patients was not altered

**Figure 3:**
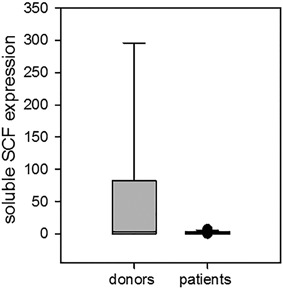
Soluble SCF expression in NK cells from patients with cancer and healthy donors. sSCF expression in CD117‐positive NK cells was assessed by qRT‐PCR as described in M&M. The expression levels were calculated as 2^−ΔΔCt^, where relative expression was determined by normalization to the beta‐actin gene expression. The mean ± SEM data for healthy donors and patients with cancer are shown: 2.81 **± **0.58 in patients (*n* = 13) versus 54 **± **32 (*n* = 9) in healthy donors (*p* = 0.08).

Evaluation of expression of soluble form of SCF in CD117‐positive NK cells revealed no statistically significant differences between healthy donors and cancer patients due to the high level of variability in healthy volunteers, although the mean values were markedly higher in healthy donors: 2.81 **± **0.58 in patients (*n* = 13) versus 54 **± **32 (*n* = 9) in healthy donors (*p* = 0.08) (Fig. 3).

sSCF expression in CD117(c‐kit)‐negative NK cells from all cancer patients was similar to that one in healthy donors and technically undetectable.

Thus, comparable down‐regulation of c‐kit and sSCF expression in NK cells suggests the alterations in c‐kit/SCF autocrine loop in NK cells.

### C‐myc expression in NK cells coincided with the c‐kit expression in donors and cancer patients

Estimation of c‐myc expression was carried out by Smart Flare method. C‐myc expression in CD117(c‐kit)‐negative fractions of NK cells from all cancer patients was similar to its expression in CD117(c‐kit)‐positive fractions; therefore, c‐myc expression was assessed in the total NK cell populations.

**Figure 4:**
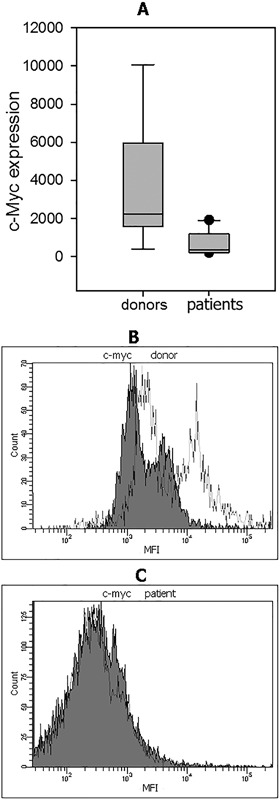
C‐myc expression in NK cells from patients with cancer and healthy donors. (A) C‐myc expression in CD117‐positive NK cells was assessed by Smart Flare as described in M&M. NK cell samples were incubated in RPMI‐1640 medium supplemented with 10% FCS in 96‐well flat‐bottom plates at 37°C, 5% CO_2_ for 20 h in the presence of Smart Flare MYC, Human, Cyanine 5. The mean ± SEM data for healthy donors and patients with cancer are shown. MFI values were 720 **±** 208 in patients (*n* = 10) versus 3596 **± **1031 in healthy donors (*n* = 9) (*p* = 0.003). (B) Representative Smart Flare results of C‐myc expression in CD117^+^ NK cells from one of nine healthy donors and (C) one of cancer patients are shown. Grey histograms—is negative control, white—is positive expression.

Surprisingly, c‐myc expression in NK cells completely coincided with the c‐kit expression in healthy donors and cancer patients. Similar to c‐kit, we revealed a significant down‐regulation of c‐myc expression in all cancer patients independently of the tumor location. For instance, MFI values were 720 **±** 208 in patients (*n* = 10) versus 3596 **± **1031 in healthy donors (*n* = 9) (*p* = 0.003) (Fig. 4).

Thereby, c‐myc expression was significantly decreased in all cancer patients independently of tumor location, stage of disease, and the presence of metastases, similar to the c‐kit expression in the same cells.

### STAT3 expression in NK cell subsets was increased in lung cancer patients, while it was decreased in cancer patients with other types of cancer

STAT3 expression was detected both in total NK cell populations and in separated CD56^bright^ and CD56^dim^ NK subsets. Revealed defects in the total population of NK cells were similar to both tested NK cell subsets. However, STAT3 expression was different in NK cells from lung cancer patients and patients with gastric, sigmoid, and colon cancer. Furthermore, STAT3 expression in NK cells was significantly different in lung cancer patients and healthy donors. Mean MFI (Mean Fluorescence Intensity) values were 5070 **± **2951 in patients (*n* = 10) versus 2523** ± **1609 in healthy donors (*n* = 13) (*p* = 0.024). It is important to note that STAT3 expression in NK cells from two patients with metastasis was higher than in patients without metastasis (6227 and 5138). The only patient who underwent three courses of chemotherapy demonstrated the higher level of STAT3 expression: 8477 and 10,112 in CD56^bright^ and CD56^dim^ NK cell subsets, respectively.

**Figure 5:**
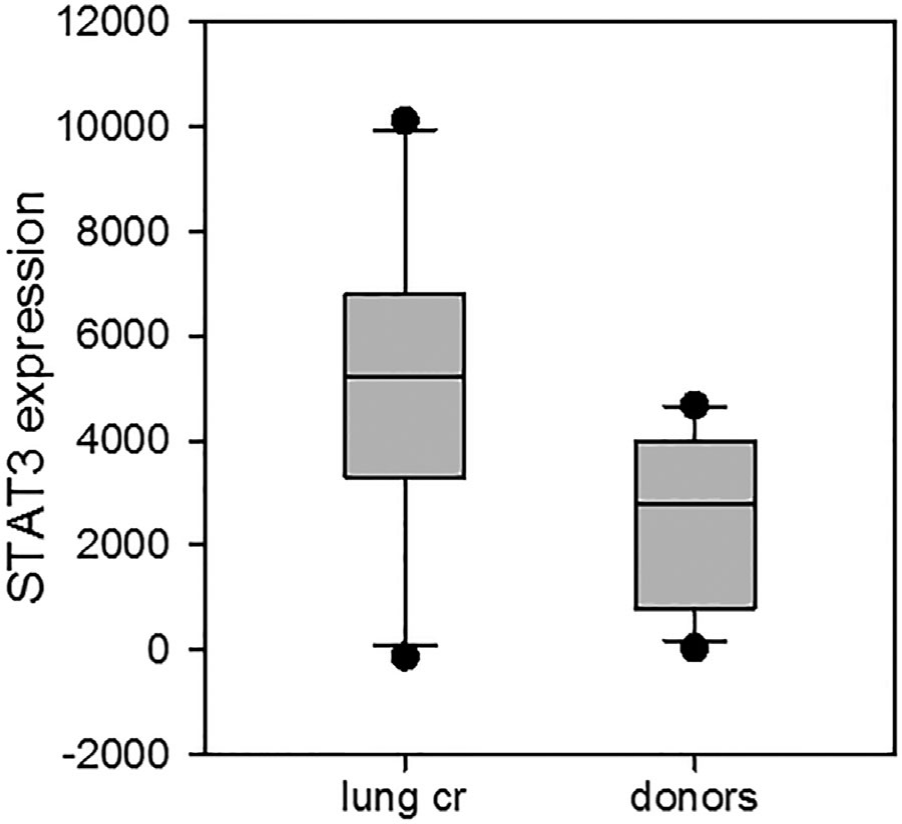
STAT3 expression in NK cells from donors, lung cancer patients, and patients with gastric, sigmoid, and colon cancer. STAT3 expression was detected by Smart Flare methodology both in total NK cell population and in separated CD56^bright^ and CD56^dim^ NK cell subsets. NK cell samples were incubated in RPMI‐1640 medium supplemented with 10% FCS in 96‐well flat‐bottom plates at 37°C, 5% CO_2_ for 20 h in the presence of Smart Flare STAT3, Human, Cyanine 5. Number of samples: lung cancer patients (*n* = 10), healthy donors (*n* = 13). Mean MFI (Mean Fluorescence Intensity) values were 5070 **± **2951 in patients versus 2523** ± **1609 in healthy donors (*p* = 0.024).

Next, although STAT3 expression in NK cells was lower in patients with gastric, sigmoid, and colon cancer in comparison to the healthy volunteers group, the difference did not reach statistical significance: MFI values were 827 **± **614 in patients (*n* = 4) versus 2523** ± **1609 in healthy donors (*n* = 13) (*p* = 0.061) (Fig. 5). However, two groups of patients demonstrated a significant difference in STAT3 expression in NK cells: MFI results were 5070 **± **2951 in lung cancer patients (*n* = 10) versus 827 **± **614 in patients with gastric, sigmoid, and colon cancer (*n* = 4) (*P* = 0.016) (Fig. 5).

Thus, in contrast to c‐kit and c‐myc oncogene expression, STAT3 expression in NK cells was significantly higher in patients with lung cancer than in healthy donors. STAT3 expression in NK cells from patients with gastric, sigmoid, and colon cancer was decreased similar to the expression of c‐kit and c‐myc oncogenes.

## Discussion

The data presented in this report, for the first time, revealed a defect in the c‐kit/SCF axis, as wells as in the c‐myc, and STAT3 expression in NK cells in patients with cancer. The c‐kit/SCF axis and c‐myc play a pivotal role in the functional activity of NK cells. C‐kit and its ligand SCF provide the important signals for survival, proliferation, migration, and cytotoxicity of NK cells. It has been shown that the generation of NK cells from the hematopoietic precursors is reduced in the absence of c‐kit signaling 5. An increased proportion of more immature and non‐cytotoxic CD56^bright^CD16^+/−^ NK cell subset in the peripheral blood of advanced breast cancer patients has been also shown 22. Myc has generally been associated with the promotion of cellular growth and proliferation 23. Conversely, the reduction of c‐myc expression has been associated with defects in cell growth and proliferation 24, 25. C‐myc regulates cell proliferation through the cell cycle machinery and the down‐regulation of c‐myc results in the growth arrest and accumulation of cells in the G0/G1 phase of the cell cycle 26. Thus, the role of c‐kit and c‐myc seems to be similar in supporting cell growth and functional activity of NK cells. Our study has revealed the disruption of both proto‐oncogenes expression in NK cells in cancer patients. The disturbances of c‐kit and c‐myc expression in NK cells shown here may be indicative for the reduced viability of NK cells, which may be one of the causal factors of decreased antitumor cytotoxicity of NK cells in cancer 27, 28, 29.

C‐myc is generally expressed at low levels in normal proliferating cells 30. Based on the central role of c‐myc in cell growth control, even slender alterations in the amount of its mRNA may have marked consequences for cell fate 31, 32, 33, 34, 35. C‐myc expression depends on the presence of growth factors and removal of signals from growth factor receptors results in immediate down‐regulation of c‐myc 36, 37. The link between the SCF‐induced cell cycle progression and c‐myc activation has been reported 38. Here, we have also noted a correlation between a decreased SCF growth factor and c‐myc expression, which might suggest that the reduced c‐myc expression is a consequence of the depressed SCF expression.

Simultaneous reduction of c‐kit and sSCF expression proves the alteration of the c‐kit/SCF autocrine loop in NK cells in cancer patients. The cause and mechanism of this phenomenon remains to be determined. The revealed thousand‐fold increase in mbSCF expression in NK cells from patients with metastatic disease is also of a great interest. The absence of reconciliation between the c‐kit and sSCF expression and the mbSCF expression prompts further speculations about the functional differences of these two forms of SCF 39. Interestingly, because the role of mbSCF in cell migratory capacity has been established 40, one can speculate that a potential damage of NK cell chemotaxis may result in the common absence of NK cells in the tumor microenvironment and, in turn, unlimited metastatic spread of tumor cells in certain types of cancer. Deterioration of migratory properties can also explain the increase of the total number of NK cells harvested from the peripheral blood of cancer patients, which was twice higher than NK cell numbers in the same volume of healthy donor blood.

Significant increase in STAT3 expression in NK cells in lung cancer patients, revealed in our studies, can be associated with c‐kit and c‐myc down‐regulation in the same cells, since STAT3 executes the downstream signaling from these proto‐oncogenes. The STAT3 transcription factor has been known for a long time as a driver of tumorigenesis and its role in the immune response to cancer has recently been also revealed. STAT3 regulates antitumor immunity, affecting innate, and adaptive immunity, cell migration to the tumor site, cytolytic function, and the secretion of cytokines and growth factors from different cells. STAT3 negatively regulates NK activation and tumor cell killing, as STAT3‐deficient NK cells generally exhibit enhanced cytolytic activity and cytokine secretion in vitro and in vivo. The molecular basis of this regulation likely involves STAT3‐mediated repression of NK cell functional molecules, such as perforin and granzyme B 41.

We can suggest a direct relationship between the alterations of c‐kit, c‐myc, and STAT3 expression in NK cells in patients with cancer. Interestingly, STAT3 expression in NK cells was increased or decreased in patients with different tumor localization, while the expression of c‐kit and c‐myc was reduced in all patients with cancer in comparison to healthy volunteers. This may denote that all cancer patients have depressed the vital NK cell activity, which may allow the initial or progressive tumorigenesis. It is also possible that the similarity in c‐kit and c‐myc expression in NK cells in all cancer patients is indicative of the general unspecific alterations in NK cells during tumor development regardless of the cancer type. In fact, tumor‐infiltrating lymphocytes, including NK cells, display constitutive STAT3 phosphorylation, which is often driven by the production of cytokines, and growth factors by malignant cells 42. Because in our studies NK cells were isolated from the peripheral blood, it is unlikely that the reason of the detected changes in NK cells is associated with the local tumor microenvironment. It is likely that the defects in NK cells are intrinsic by nature. Actually, multiple mutations affecting the immune response have been suggested as a potential reason for the development of breast cancer 43. Therefore, further investigation of possible changes in c‐kit, c‐myc and STAT3 expression and function in other types of immune cells in human cancer are important. For instance, c‐kit/SCF signaling pathway is known to regulate function of dendritic cells 44, 45. Dendritic cell maturation from the bone‐marrow progenitors is disturbed by c‐kit blockade 46, which may be a reason for dendritic cell maturation defects in cancer 47, 48, 49.

In summary, our data reveal an abnormal expression of c‐myc, с‐kit, and STAT3 expression in NK cells from the peripheral blood of patients with different types of cancer. However, it is unclear whether these alterations are induced by the tumor‐associated factors systemically or represent intrinsic defects in NK cells that precede the tumor development. Future dynamics and genetic studies are needed to clarify this issue and explain the new findings described here.

## Authors’ Contributions

G.K. Zakiryanova, N.N. Nakisbekov, M.R. Shurin contributed in Conception and design, G.K. Zakiryanova contributed in the Acquisition of data (acquired and managed patients, provided facilities), E.Kustova, N. T. Urazalieva, A. Amirbekov, E. T. Baimuchametov contributed in the development of methodology, G.K. Zakiryanova contributed in the analysis and interpretation of data (e.g., statistical analysis, biostatistics, computational analysis), G.K. Zakiryanova, M.R. Shurin contributed in the writing, review, and/or revision of the manuscript: N.N. Nakisbekov contributed in the administrative, technical, material support, and G.K. Zakiryanova contributed in the Study supervision.

## Conflicts of Interest

No potential conflicts of interest were disclosed.
